# Acute Hepatopancreatic Necrosis Disease (AHPND) related microRNAs in *Litopenaeus vannamei* infected with AHPND-causing strain of *Vibrio parahemolyticus*

**DOI:** 10.1186/s12864-018-4728-4

**Published:** 2018-05-08

**Authors:** Zhihong Zheng, Jude Juventus Aweya, Fan Wang, Defu Yao, Jingsheng Lun, Shengkang Li, Hongyu Ma, Yueling Zhang

**Affiliations:** 1 Department of Biology and Guangdong Provincial Key laboratory of Marine Biotechnology, Shantou University, Shantou, 515063 Guangdong China; 2 Department of Biology, School of Science, Shantou University, Shantou, 515063 Guangdong China

**Keywords:** *Litopenaeus vannamei*, MicroRNAs, AHPND, *Vibrio parahemolyticus*

## Abstract

**Background:**

Acute hepatopancreatic necrosis disease (AHPND) has emerged as a major debilitating disease that causes massive shrimp death resulting in substantial economic losses in shrimp aquaculture. Given that several diseases and infections have been associated with microRNAs (miRNAs), we conducted a comparative transcriptomic analysis using the AHPND (VA) and non-AHPND (VN) strains of *Vibrio parahemolyticus* to identify miRNAs potentially involved in AHPND pathogenesis in *Litopenaeus vannamei*.

**Results:**

A total of 83 miRNAs (47 upregulated and 36 downregulated) were significantly differentially expressed between the VA and VN challenged groups, while 222 target genes of these miRNAs were predicted. Functional enrichment analysis revealed that the miRNAs target genes were involved in multiple biological processes including metabolic pathways, amoebiasis, *Vibrio cholerae* infection etc. Finally, interaction network and qPCR (Real-time Quantitative PCR) analysis of 12 potential key AHPND-related miRNAs and their predicted target genes, revealed their possible involvement in modulating several immune-related processes in the pathogenesis of AHPND.

**Conclusions:**

We have shown using comparative transcriptomic analysis, miRNAs and their target genes that are responsive to AHPND *V. parahemolyticus* infection in shrimp, therefore suggesting their possible role in defense response to AHPND *V. parahemolyticus* infection.

**Electronic supplementary material:**

The online version of this article (10.1186/s12864-018-4728-4) contains supplementary material, which is available to authorized users.

## Background

MicroRNAs (miRNAs) are endogenous non-coding small RNA (sRNA) [1], which act by negatively modulating gene expression at the post-transcription level [2], via repression of messenger RNAs (mRNA) translation, destabilization and degradation of transcript by binding to the 3′ UTR (Untranslated Region) of the target mRNAs [3]. These molecules act as important regulators in diverse life processes, including cellular proliferation, apoptosis and immunity [4].

*Litopenaeus vannamei* is one of the largest aquaculture shrimp species in the world with huge production and economic benefits [5]. The shrimp aquaculture industry has however been saddled with diseases and infections, which cause significant losses in shrimp production. A number of studies in mammals have suggested that host miRNAs are key regulators of virus-host interactions during an infection [6–8], while in different crustaceans miRNAs have been identified to be involved in the response to some diseases and infections. For instance, Li et al. identified some microRNAs involved in the response of Chinese shrimp *Fenneropenaeus chinensis* to white spot syndrome virus (WSSV) infection [9]. Similarly, Zhu et al. screened 55 host miRNAs which responded to *Vibrio alginolyticus* infection in shrimp *Marsupenaeus japonicas* [10], while Ou et al. identified 33 dysregulated miRNAs in crayfish *Procambarus clarkii* upon challenge with *Spirolasma eriocheiris* [11]. In recent years, an emerging shrimp disease called acute hepatopancreatic necrosis disease (AHPND), brought about significant losses in aquaculture shrimp production, resulting in great economic losses. The pathogenesis of AHPND was first though to be related to the environment, wherein a multiple and complex interplay of factors including bacteria [12, 13], virus [14, 15] as well as other environmental factors coexist [16, 17]. Of these factors, the *V. parahemolyticus* with photorhabdus insect-related (Pir) toxins was finally implicated as the most probable major pathogenic factor [18],as this bacteria is capable of producing the characteristic symptoms of AHPND [19]. However, it is still largely unknown how shrimp miRNAs respond to AHPND *V. parahaemolyticus* infection.

Our unpublished shrimp hemocytes comparative transcriptome data (GenBank accession number: PRJNA385392) revealed a large number of differentially expressed genes following challenge of shrimps with AHPND and non-AHPND *V. parahaemolyticus*. Based on this, we sought to explore in this study the responsive miRNAs that might regulate the expression of these genes in shrimps infected with AHPND and non-AHPND *V. parahaemolyticus.* This study therefore provides a transcriptome-based analysis of miRNAs and their target genes in *L. vannamei* challenged with AHPND *V. parahaemolyticus*, thereby laying the framework for further exploring miRNAs and their roles in regulating host responses to AHPND.

## Methods

### Animal challenge and hemocytes collection

Healthy adult shrimps (*L. vannamei*) (weighing about 10~ 12 g) were obtained from Shantou Huaxun Aaquatic Product Corporation (Shantou, Guangdong, China) and acclimatized to laboratory conditions for 3–5 days before challenge experiments. Bacteria (non-AHPND and AHPND *V. parahaemolyticus*, kind gift from Prof. Lo, National Cheng Kung University, Taiwan, China) at the logarithmic growth phase were cultured in Luria-Bertani media (tryptone: 10 g/L, yeast extract: 5 g/L, NaCl 30 g/L) at 28 °C with shaking, counted and diluted to the appropriate colony forming units (CFU) with 0.65% normal saline. After the period of laboratory acclimatization, shrimps were randomly divided into three experimental groups with at least 50 shrimps per group per container. The VN group denotes shrimps injected with 50 μl of non-AHPND *V. parahaemolyticus* (1.0 × 10^5^ CFU/ml), VA group represent shrimps injected with 50 μl of AHPND *V. parahaemolyticus* (1.0 × 10^5^ CFU/ml), while the NS group denotes shrimps injected with 50 μl of normal saline (0.65% NaCl, Xilong Scientific, Shantou, China). Hemolymph from ten randomly selected shrimps per group was drawn at 24 h post infection (hpi) through the pericardial sinus into an equal volume of cold anti-coagulant buffer (NaCl 450 mM; KCl 10 mM; EDTA-Na_2_ 10 mM; HEPES 10 mM. pH 7.0), centrifuged immediately at 800 g for 10 min at 4 °C to collect the hemocytes for RNA extraction. Three biological replicates per treatment group were prepared for the sRNA library construction.

### sRNA library and high-throughput sequencing

Total RNA including sRNA was extracted from hemocytes using the *mirVana* miRNA Isolation Kit (Ambion, Austin, TX, USA) according to the manufacturer’s instructions. The concentration and purity of the total RNA was quantified with a NanoDrop 2000 spectrophotometer (Nano-drop Technologies, Wilmington, DE), while the total RNA integrity was evaluated using an Agilent 2100 Bioanalyzer system (Agilent Technologies, Palo Alto, California.) with a RNA integrity number (RIN) > 8.5. Only high quality RNA samples were used for constructing the cDNA libraries. Before use, all samples were diluted to a final concentration of 200 ng/μL. Sequencing libraries were generated with the TruSeq® smallRNA Sample Preparation kit (Illumina, San Diego, USA). The concentration of the cDNA libraries was determined with the KAPA Library Quantification Kit (KAPA Biosystems) followed by sequencing on an Illumina HiSeq2000 sequencer (Illumina, San Diego, USA) employing single-end sequencing strategy with 1 × 50-bp read length. Sequencing work was commissioned by Beijing Genomics Institute (BGI, Wuhan, China). The raw sequencing data from this study have been deposited at GenBank with accession number PRJNA414944.

### Bioinformatic analysis of miRNA data

The raw miRNA data was first cleaned by removing 5′ primer contaminants, no insert tags, oversized insertions, low quality reads and polyA tags. Length distribution analysis of the clean reads was then carried out before miRNA prediction. To remove rRNA-, scRNA-, snoRNA-, snRNA-, and tRNA-associated tags, reads were aligned with GenBank and Rfam reference databases using Blast or Bowtie. The obtained sequences were then mapped against our *L. vannamei* hemocyte transcriptome (GenBank accession: PRJNA385392) to remove degraded mRNA and reads of unannotated transcripts. Following this, reads were then mapped against the miRNA database (miRBase 21), to obtain matching miRNA sequences conserved in *L. vannamei*. The obtained miRNAs sequences were used for family classification and annotation with the miRClassify web server [20]. Finally, the sRNAs that did not map to other miRNA sequences were regarded as novel miRNAs [21]. Differential expression of the identified miRNAs was computed according to the DESeq method [22], while the Bayesian approach was applied for the statistical analysis of the data [23].

### Target genes prediction and miRNA-mRNA interaction network analysis

To test for significant associations between genes, miRNAs, pathways and GO terms, the prediction software TargetScan and RNAhybrid, together with the differentially expressed genes in the *L. vannamei* hemocytes transcriptome data (Genebank accession numbers: PRJNA385392) were used. The relatedness or anticorrelation of the expression profiles of both the target genes and the associated miRNAs (downregulated miRNAs – upregulated mRNAs and upregulated miRNAs – downregulated mRNAs) based on the output of TargetScan or/and RNAhybrid were considered. Next, all the target genes obtained were subjected to GO and KEGG analysis. Finally, Cytoscape (version 3.5.1), an open source software platform was used to construct the interaction network between the miRNAs and mRNAs.

### Real-time quantitative PCR (qPCR) analysis

To validate the miRNA-seq data, 12 VA versus VN significant differentially expressed miRNAs were selected for qPCR analysis. One microgram (1 μg) of the high quality total RNA samples, extracted using the *mirVana* miRNA Isolation Kit (Ambion, Austin, TX, USA), from each treatment were polyadenylated with poly (A) followed by first-strand cDNA synthesis with the TransScript miRNA First-Strand cDNA Synthesis SuperMix (TransGen Biotech, Beijing, China) kit following the manufacturer’s instruction. The forward miRNA primers (Table 1), designed based on the mature miRNA sequence, were used with a universal primer (supplied with TransScript miRNA First-Strand cDNA Synthesis SuperMix Kit, TransGen Biotech, Beijing, China) to amplify the mature miRNAs. The qPCR assay was performed at the following cycling conditions: one cycle at 95 °C for 10 min and 45 cycles of 95 °C for 15 s and 60 °C for 30 s, with the TransStart Tip Green qPCR SuperMix (TransGen Biotech, Beijing, China) kits on Roche LightCycler 480 detection system (Roche, Basel, Switzerland). U6 snRNA (small nuclear RNA) was used as an internal control (see Table 1 primers sequence). All the reactions were performed in replicates and with three biological replicates. The relative miRNA expression levels were calculated using the 2^-∆∆CT^ method. Statistical analysis was performed using a two-tailed unpaired Student’s t-test with *p* < 0.05 considered statistically significant.

**Table 1:** Nucleotide sequence of primers used in this study

Primer name	Primer sequence (5′ ➔ 3′)
U6-F	CTCGCTTCGGCAGCACA
U6-R	AACGCTTCACGAATTTGCGT
mja-miR-6489-5p	GGCACCGGACTGGCGCCCTT
dre-miR-107b	AGCAGCATTGTACAGGGCTTT
bta-miR-4286	ACCCCACTCCTGGTACC
tch-miR-210	CTGTGCGTGTGACAGCGGCTGA
hsa-miR-211-5p	TTCCCTTTGTCATCCTTCGCCT
sha-miR-10b	TACCCTGTAGAACCGAAT
dpu-miR-193	TACTGGCCTGCTAAGTCCCAAA
bta-miR-22-5p	AGTTCTTCAGTGGCAAGCTTTA
bmo-miR-9c-5p	TCTTTGGTATCCTAGCTG
bmo-miR-278-5p	CCGGACGAACTTCCCAGCTCGG
bbe-miR-210-5p	CTGTGCGTGTGACAGCGGCTGA
age-miR-127	TCGGATCCGTCTGAGCTTGGCT

## Results

### Overview of miRNA transcriptome data

**Fig. 1:**
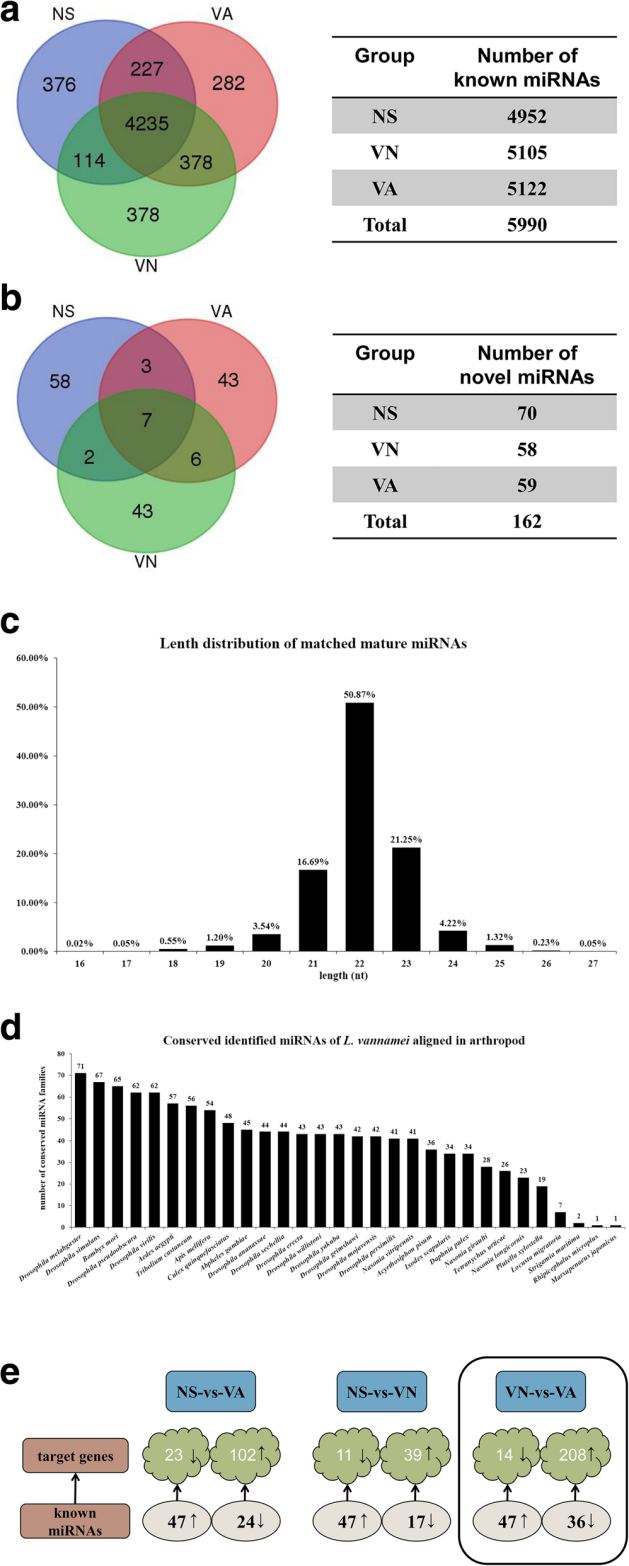
Characteristics of identified miRNA from *L. vannamei*. Summary of known (**a**) and predicted novel (**b**) miRNAs from hemocyte miRNA libraries, with samples from AHPND *V. parahaemolyticus* (VA), non-AHPND *V. parahaemolyticus* (VN) and normal saline (NS) challenged shrimps. **c** Mature miRNAs from sRNAs matched to miRBase. **d** MiRNA family analysis of conserved miRNAs. The Y-axis indicated the number of conserved miRNA families of *L. vannamei* aligned in arthropods. **e** Diagram summarizing differentially expressed miRNAs (known and novel) and their predicted target genes. Target genes were obtained from in-house hemocyte transcriptome data. Boxed shows data comparison between VN and VA

To characterize the AHPND related miRNAs in shrimp, nine sRNA libraries of shrimp hemocytes from three independent experiments were constructed, with three treatment groups denoted NS (normal saline), VN (non-AHPND *V. parahemolyticus*), and VA (AHPND *V. parahemolyticus*). High-throughput Illumina sequencing was performed on the sRNA libraries generating a total of 107,159,619 raw sequencing reads. Further filtering to remove rRNA (ribosomal RNA), scRNA (small cytoplasmic RNA), snoRNA(small nucleolar RNA), snRNA, tRNA (transfer RNA), and degraded mRNA fragments, was carried out by aligning with GenBank and Rfam reference databases as well as with our own hemocytes transcriptome data. After data filtering, 98,629,518 clean reads were left representing 92.04% of total raw reads (Table 2). The resulting reads were then mapped against the miRBase 21.0 database to obtain the matching shrimp miRNA homologs. A total of 4952, 5105 and 5122 known miRNA homologs from NS, VN and VA respectively were obtained, giving a grand total of 5990 identified known miRNAs (Fig. 1a). Of the total identified known miRNAs, 4235 were common to all treatment groups while 376, 378 and 282 miRNAs were unique to NS, VN and VA respectively (Fig. 1a). MiRNA family cluster analysis with other species yielded 70, 58 and 59 miRNAs as probable novel miRNAs from NS, VN and VA respectively, giving a total of 162 predicted novel miRNAs (Fig. 1b). Size distribution analysis of the 5990 identified known miRNAs (Fig. 1c) showed that 96.57% were between 20 and 24 nt in length, which is typical of miRNAs [24].

**Table 2:** Summary of clean and raw reads

Type	Count (Clean reads/Total raw reads)	Percent (%)
NS1	10,159,067/11351071	89.62%
NS2	10,389,676/11327961	91.87%
NS3	11,073,782/12274097	90.37%
VN1	11,365,725/11929741	95.41%
VN2	10,955,348/11876335	92.38%
VN3	11,073,782/12274097	90.37%
VA1	10,939,945/11699373	93.67%
VA2	11,379,457/12182226	93.59%
VA3	11,292,736/12244718	92.40%
Total	98,629,518/107159619	92.04%

Next, the identified miRNAs were classified into 103 families (Additional file 1: Table S1), with most of the miRNA families highly conserved in arthropods. For instance, 71 were found in *Drosophila melabgaster*, 67 in *Drosophila simulans*, 65 in *Bombyx mori*, 62 in *Drosophila pseudoobscura*, but only 35 found in crustaceans (i.e., 34 found in *Daphnia pulex* and 1 in *Marsupenaeus japonicas*). Figure 1d shows the family distribution of the identified miRNAs in arthropods.

To identify the *V. parahemolyticus* responsive miRNAs, the DESeq method [22], which requires biological replicate samples, with a fold change > 2 for upregulated miRNAs and fold change ≤ 0.5 for downregulated miRNAs were used [22]. With these fold change cutoffs, 71 dysregulated miRNAs were identified upon AHPND *V. parahemolyticus* challenge while 64 dysregulated miRNAs were identified upon non-AHPND *V. parahemolyticus* challenge. When the non-AHPND (VA) and AHPND (VN) infection groups were compared, 47 upregulated and 36 downregulated miRNAs were identified (Fig. 1e).

### Target genes prediction and functional analysis of differentially expressed miRNAs

Given that miRNAs negatively regulate mRNA expression [25], we went on to combine this miRNA transcriptome data with our our previous shrimp *L. vannamei* mRNA transcriptome data (GenBank accession number: PRJNA385392) so as to identify the miRNA target genes. Two target gene prediction software, i.e., Targetscan and RNAhybrid were used to obtain all the differentially expressed miRNA target genes. Next, target genes whose expressions were inversely correlated or with reciprocal expression pattern with their corresponding miRNAs were extracted. Using these target prediction criteria, 125 and 50 target genes were identified for the AHPND and non-AHPND *V. parahemolyticus* infected groups, respectively. When the predicted target genes for VA and VN were compared, 208 upregulated and 14 downregulated targets genes were identified to be candidate AHPND-related miRNA target genes (Fig. 1e and Additional file 2: Table S2).

**Fig. 2:**
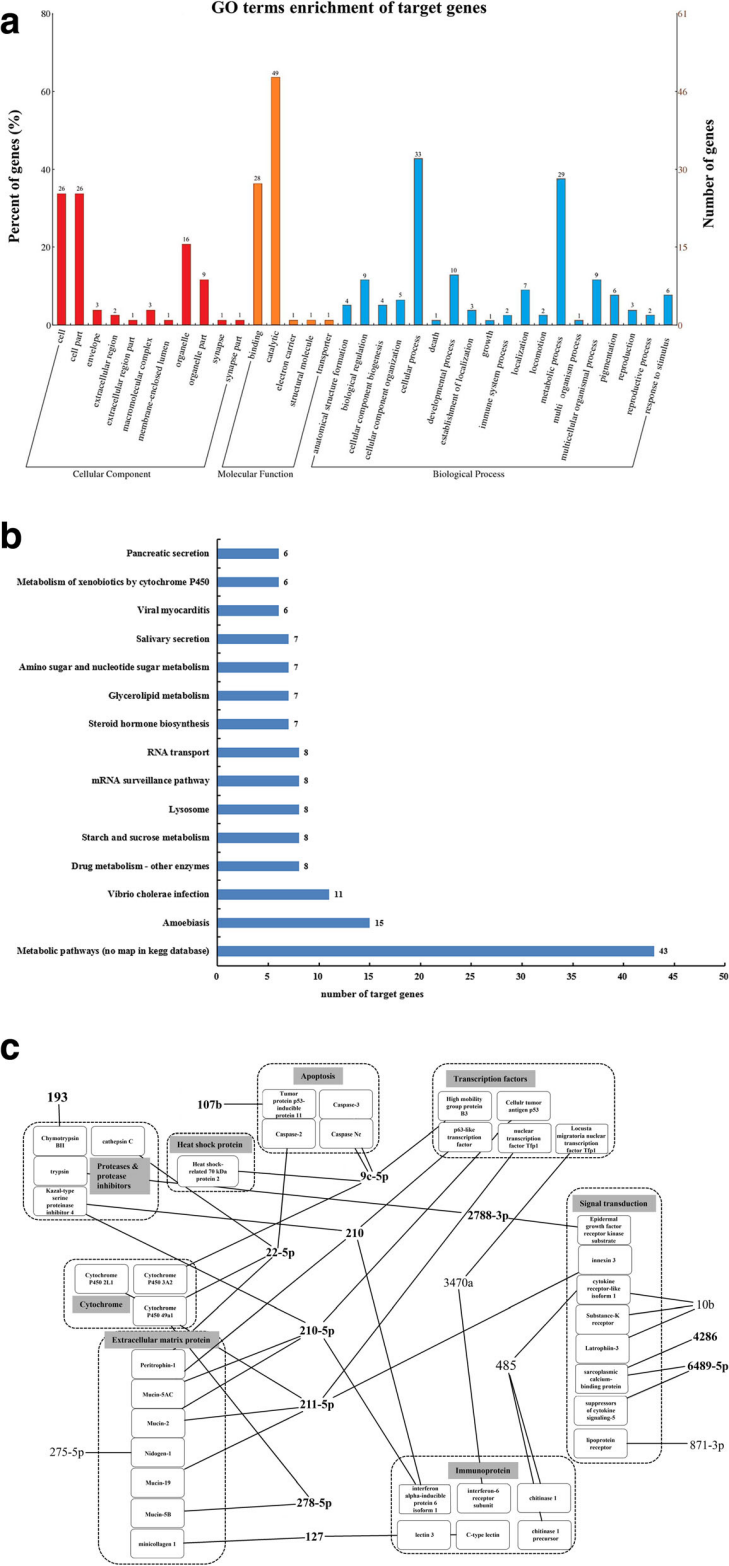
Function annotation of predicted target genes between VN and VA treated group. **a** GO enrichment of target genes. **b** KEGG analysis of target genes. **c** Interaction network analysis between miRNAs and their target genes. MiRNAs in black bold type were used for qPCR validation

To gain an insight into how miRNAs affect AHPND, the AHPND-related miRNA target genes identified were categorized by GO and KEGG analyses. For the highly represented terms in each GO category, 77 DEGs in the VN versus VA treated groups were enriched in cellular component (89), molecular function (80), and biological process (137). In terms of GO function annotation, most of the DEGs were enriched in cell (26), cell part (26), catalytic (49), binding (28), cellular process (33), and metabolic process (29) (Fig. 2a). KEGG analysis was further performed to so as to identify the pathways involved in shrimps’ response to AHPND. As shown in Fig. 2b, the five most highly represented pathways include metabolic pathways (43), amoebiasis (15), *Vibrio cholerae* infection (11), drug metabolism - other enzymes (8), starch and sucrose metabolism (8).

Among the 222 predicted AHPND-related miRNA target genes, we excluded uncharacterized or unannotated genes, leaving with 124 genes, after which 38 immune related genes and their corresponding miRNAs (17 miRNAs) were chosen for further evaluation. Next, we went on to construct network interaction between these 17 miRNAs and their putative target genes (Fig. 2c and Table 3). It was observed that 14 downregulated miRNAs had 35 upregulated target genes while 3 upregulated miRNAs had 3 downregulated target genes (Fig. 2c). Broadly, these 38 immune-related genes encode for eight categories of proteins including 4 apoptosis related proteins, 1 heat shock protein, and 6 immunoproteins (Fig. 2c and Table 3). The network interaction analysis showed some possible interaction between the AHPND-related miRNAs and HSP70 (Fig. 2c).

**Table 3:** Significant differentially expressed miRNAs between VN and VA treated groups and their predicted targets

miRNA	Log2 fold change (VA/VN): miRNA transcriptome	Log2 fold change (VA/VN): qPCR	Targets	Log2 fold change (VA/VN): target genes	Annotation
bbe-miR-210-5p	−2.870407322	−1.517940555	Unigene5114_All	1.060349045	p63-like transcription factor
			CL3703.Contig2_All	1.088996766	interferon alpha-inducible protein 6 isoform 1
			Unigene10324_All	1.201529506	Mucin-5 AC
			Unigene444_All	1.267391152	Mucin-2
			Unigene7288_All	2.244651906	Kazal-type serine proteinase inhibitor 4
age-miR-127	−2.588007591	−1.649872672	CL878.Contig1_All	1.4821571	lectin 3
			CL793.Contig1_All	1.830735508	C-type lectin
			Unigene711_All	1.769299871	minicollagen 1
hsa-miR-211-5p	−5.092799743	−1.708656758	CL1084.Contig1_All	1.166678298	Cytochrome P450 2 L1
			Unigene23044_All	1.357617048	Mucin-19
			Unigene444_All	1.267391152	Mucin-2
			Unigene20688_All	1.711317404	nuclear transcription factor Tfp1
			CL1920.Contig3_All	1.465320468	innexin 3
tch-miR-210	−2.815959538	−1.589144004	Unigene5114_All	1.060349045	p63-like transcription factor
			CL3703.Contig2_All	1.088996766	interferon alpha-inducible protein 6 isoform 1
			Unigene10324_All	1.201529506	Mucin-5 AC
			Unigene444_All	1.267391152	Mucin-2
			Unigene7288_All	2.244651906	Kazal-type serine proteinase inhibitor 4
bmo-miR-278-5p	−5.70048232	−1.446914278	Unigene10827_All	1.273096701	cytochrome P450 49a1
			Unigene6914_All	1.242144205	Mucin-5B
bta-miR-22-5p	−4.70048232	−1.998632464	Unigene1405_All	1.881244868	Caspase-2
			Unigene1487_All	5.962236179	Peritrophin-1
			Unigene10827_All	1.273096701	cytochrome P450 49a1
			Unigene7890_All	1.269494038	cathepsin C
bmo-miR-9c-5p	−4.802020347	−1.286244383	Unigene20818_All	1.499249658	Caspase Nc
			Unigene5907_All	12.02859678	Heat shock-related 70 kDa protein 2
			Unigene24983_All	5.12621573	High mobility group protein B3
			CL183.Contig3_All	1.643990119	Caspase-3
			Unigene21910_All	1.036005418	Cytochrome P450 3A2
dpu-miR-193	−1.274473703	−1.428703251	Unigene18431_All	2.401408994	Chymotrypsin BII
dre-miR-107b	3.106872602	1.737363291	CL1260.Contig2_All	−1.153013031	Tumor protein p53-inducible protein 11
mja-miR-6489-5p	1.182514886	5.323473519	CL1919.Contig1_All	−1.964210604	sarcoplasmic calcium-binding protein
			Unigene15684_All	−1.244264787	suppressors of cytokine signaling-5
bta-miR-4286	4.943373869	1.464858155	CL1919.Contig1_All	−1.964210604	sarcoplasmic calcium-binding protein
sha-miR-10b	−1.161907157	−1.211066559	Unigene2751_All	1.405570272	Substance-K receptor
			Unigene22975_All	1.035210199	Cytokine receptor
			Unigene15784_All	1.371799521	Latrophilin-3

### Validation of potential key AHPND-related miRNAs using qPCR

**Fig. 3:**
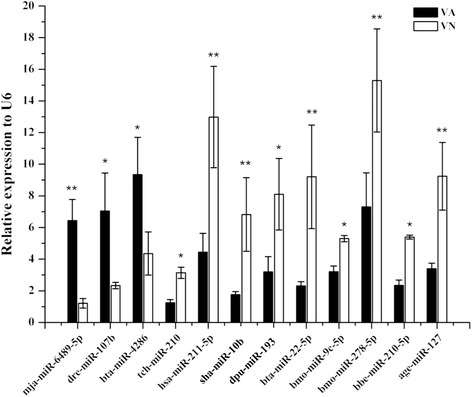
Validation of differentially expressed miRNAs between VN and VA treated group by qPCR. Real-time qPCR was used to analyze the relative expression of 12 miRNAs and normalized to U6 snoRNA. Data presented is average of three independent experiments. The error bar represents the S.D. of the mean (*n* = 3). The statistical significance was calculated using Student’s t-test with *p* < 0.05 considered statistically significant compared with NS control

To further substantiate the miRNA data obtained, 12 key putative AHPND-related miRNAs were chosen from the network analysis (Fig. 2c) for validation using qPCR, with U6 snRNA as the internal control. Three miRNAs, mja-miR-6489-5p, dre-miR-107b, and bta-miR-4286 were significantly upregulated in VA as compared to VN, while nine miRNAs including tch-miR-210, sha-miR-10b, and dpu-miR-193 were significantly downregulated (Fig. 3). The expression levels of all the miRNAs were expressed relative the NS group. The qPCR results were generally consistent with the miRNA transcriptome data, as shown in Table 3, where data for upregulated and downregulated miRNAs showed a similar trend.

## Discussion

Acute Hepatopancreatic Necrosis Syndrome (AHPNS) or Early Mortality Syndrome (EMS), which has now been shown to have bacterial aetiology [26], has caused significant losses in shrimp aquaculture in recent years. Given their role as one of the main post-transcriptional regulatory factors, miRNAs are implicated in various physiological and pathophysiological processes including differentiation, metabolism, signal transduction, stress responses, and immunity [27]. In the last couple of years, multiple functions of some crustaceans’ miRNAs have been reported. For instance, it has been shown that shrimp miR-1000 could target the 3′-untranslated region (3’UTR) of the p53 gene to regulate apoptosis and virus infection via the p53 pathway [28]. Similarly, miR-9 was shown to be upregulated or downregulated when shrimp apoptosis was induced or inhibited, suggesting that miR-9 participated in the apoptosis pathway by acting as a modulator [29]. Similarly, shrimp miR-1959 mediates a feedback loop, which could directly regulate the NFκB/IκB complex by targeting the 3’-UTR of cactus gene [30].

Although *L. vannamei* is the most widely cultured shrimp species in the world, and miRNAs have been extensively studied in the last couple of years, there is still limited information on shrimp miRNAs. Ruan et al. were the first to isolate 35 miRNAs from *Marsupenaeus japonicas*, and showed that the expression levels of 22 miRNAs changed upon WSSV infection [31]. Similarly, Huang et al. identified 63 shrimp miRNAs in WSSV-infected shrimp at different time points post-infection [32]. Since AHPND has had devastating effects on shrimp aquaculture couple with the conflicting accounts of the disease etiology and pathogenesis, we sought to gain a better understanding of the pathogenesis and progression of AHPND. To do this, we went about to determine the miRNAs that might be differentially expressed following infection of shrimps with AHPND and non-AHPND strains of *V. parahaemolyticus*. MiRNA transcriptome analysis showed 47 upregulated and 36 downregulated miRNAs between VA and VN. Twelve differentially expressed and potential AHPND-related miRNAs including miR-210, miR-10b and miR-193 were selected and validated by qPCR. Interestingly, miR-210 had previously been reported in eel, *Branchiostoma belcheri*, and was shown to increase in expression upon *V. parahaemolyticus* infection [33], while miR-10b was shown to be upregulated upon lipopolysaccharide (LPS) treatment [34]. It thus seems miR-210 and miR-10b are typical bacterial induced miRNAs, as our KEGG analysis showed that the predicted target genes of the 12 potential AHPND-related miRNAs, were mainly enriched in the *Vibrio cholerae* subcategory. For miR-193, it had previously been reported to be associated with WSSV infection [35], and was shown to be upregulated upon low virulent WSSV infection [36]. While miR-210, miR-10b and miR-193 were shown in previous studies to be upregulated, their expression in this study was observed to be downregulated (VA relative to VN), suggesting that probably the expression of these miRNAs was suppressed so as to increase the expression or function of their associated target genes; a possible implication of their importance in shrimp immune defense.

Since the complete genome sequence of *L. vannamei* is currently unavailable, we used our previous *V. parahaemolyticus* infected shrimp mRNA transcriptome data (GenBank accession number: PRJNA385392) to help us identify the AHPND *V. parahaemolyticus* specific responsive genes, which are the target genes of the identified miRNAs. Based on this in-house database, p53 was predicted as the target gene of miRNA-210 and miRNA-210-5p (downregulated in virulent *V. parahaemolyticus* infected shrimp). Indeed, miRNA-210 has been reported to suppress p53 in cancer cell line [37]. Thus, the upregulation of p53 expression seems to suggest that apoptosis was induced in shrimp hemocytes upon AHPND *V. parahaemolyticus* infection [28]. At the same time, caspase 2, a miRNA-22-5p predicted target gene, was upregulated in AHPND *V. parahaemolyticus* infected shrimp. Since caspase 2 is anti-apoptosis or prosurvival related, its upregulation might be a response to counter the apoptosis induction [38, 39]. The strong modulation of apoptosis seems to induce a higher metabolic rate and consequently a stronger immune responses in AHPND *V. parahaemolyticus* infected shrimp. For example, miRNA-278-5p, miRNA-22-5p, miRNA-211-5p, and miRNA-9c-5p whose predicted target genes are cytochrome P450, 2 L1, 49a1, and 3A2, were downregulated in AHPND *V. parahaemolyticus* infected shrimp, suggesting the involvement of energy metabolism [40] in AHPND *V. parahaemolyticus* infected shrimp. Since cytochrome P450 is involved in the metabolism of insecticides [41], the dysregulation by these miRNAs suggests some response to the bacterial Pir toxin [42, 43]. In response to the AHPND *V. parahaemolyticus* infection, shrimps mounted a strong immune response, as there was downregulation of miRNA-127, miRNA-193, and miRNA-10b, whose target genes are C-type lectin, Chymotrypsin BII, and cytokine receptor-like isoform 1, which are immune-related. There was also the downregulation of miR-211, which was predicted to target the Mucin2 gene. MiR-211 has been reported as a potential suppressor of MUC4 and shown to inhibit cervical cancer cell invasion and epithelial-to-mesenchymal transition (EMT) [44]. Given that Mucin glycoproteins are among the main components of the first barrier that bacteria encounter in an invasion [45, 46], it thus suggest the downregulation of miR-211 in AHPND *V. parahaemolyticus* infected shrimp might be an immune defense mechanism employed by hemocytes by enhancing the expression of Mucin. Apart from these, network interaction analysis revealed some possible interaction between the AHPND-related miRNAs and HSP70, which is in tandem with a recent study which have reported that HSP70 and HSP90 are involved in the tolerance of shrimp to AHPND-causing strain of *V. parahaemolyticus* [47]. Collectively, these results suggest that the dysregulation of the 12 miRNAs identified following the infection of shrimps with AHPND *V. parahaemolyticus* was an immune defense mechanism, since this was to enhance the level of metabolism, increase the expression of immune related genes, as well as modulate cellular processes such as apoptosis.

## Conclusions

In this study, we observed the expression of 5990 shrimp miRNAs following *V. parahaemolyticus* infection, among which 12 potential AHPND-related miRNAs were identified. Furthermore, the predicted target genes of these miRNAs are involved in several immune-related processes. This work provides useful information for future research on AHPND and shrimp immune defense in terms of the relevant related miRNAs and their target genes.

## Additional files

Additional file 1: **Table S1.** Conserved miRNA families analysis of *L. vannamei*. (XLS 76 kb)

Additional file 2: **Table S2.** The potential targets of AHPND related miRNAs. (XLS 63 kb)

## Abbreviations

AHPND: Acute Hepatopancreatic Necrosis Disease
EMS: Early Mortality Syndrome
hpi: Hours post infection
LPS: Lipopolysaccharide
miRNAs: microRNAs
mRNA: Messenger RNAs
NS: Normal Saline
Pir toxin: Photorhabdus insect-related toxin
qPCR: Real-time Quantitative PCR
rRNA: Ribosomal RNA
scRNA: Small cytoplasmic RNA
snoRNA: Small nucleolar RNA
snRNA: Small nuclear RNA
sRNA: Small RNA
tRNA: Transfer RNA
UTR: Untranslated Region
VA: AHPND *Vibrio parahaemolyticus*
VN: Non-AHPND *Vibrio parahaemolyticus*
WSSV: White Spot Syndrome Virus
